# Advances in Polyhydroxyalkanoate (PHA) Production

**DOI:** 10.3390/bioengineering4040088

**Published:** 2017-11-02

**Authors:** Martin Koller

**Affiliations:** 1 Institute of Chemistry, University of Graz, NAWI Graz, Heinrichstrasse 28/III, 8010 Graz, Austria; martin.koller@uni-graz.at; Tel.: +43-316-380-5463; 2 ARENA—Association for Resource Efficient and Sustainable Technologies, Inffeldgasse 21b, 8010 Graz, Austria

**Keywords:** bacteria, copolyester, feedstocks, fermentation, haloarchaea, metabolism, mixed microbial cultures, polyhydroxyalkanoate, strain selection, process engineering, processing, pure culture, sustainability, waste streams

## Abstract

This editorial paper provides a synopsis of the contributions to the *Bioengineering* special issue “Advances in Polyhydroxyalkanoate (PHA) Production”. It illustrates the embedding of the issue’s individual research articles in the current global research and development landscape related to polyhydroxyalkanoates (PHA). The article shows how these articles are interrelated to each other, reflecting the entire PHA process chain including strain selection, metabolic and genetic considerations, feedstock evaluation, fermentation regimes, process engineering, and polymer processing towards high-value marketable products.

## 1. Introduction

Nowadays, it is generally undisputed that we need alternatives for various fossil-resource based products such as plastics, which make our daily life comfortable. Plastics, a group of polymeric materials not produced by Mother Nature, are currently produced at increasing quantities, now in a magnitude of about 300 Mt per year [1]. Such well-established plastics are used in innumerable fields of application, such as packaging materials, parts in the automotive industry, biomedical devices, electronic parts, and many more. Despite their high impact in facilitating our everyday life, current plastic production faces essential shortcomings, such as the ongoing depletion of fossil resources, growing piles of waste consisting of non-degradable full-carbon backbone plastics, and elevated CO_2_ and toxin levels in the atmosphere caused by plastic incineration [2,3].

To overcome the abovementioned evils, the last decades were devoted to find a way out of the fatal “Plastic Age” we live in today. Switching from petrol-based plastics to bio-alternatives with plastic-like properties, which are based on renewable resources, and which can be subjected towards biodegradation and composting, is regarded as one of these exit strategies [3]. Polyhydroxyalkanoates (PHA), microbial storage materials produced by numerous prokaryotes, are generally considered auspicious candidates to replace traditional plastics in several market sectors, such as the packaging field, or in biomedical applications [4,5,6,7]. To make PHA competitive, they must compete with petrol-based plastics both in terms of quality and economic aspects. Quality improvement of PHA-based materials is currently achieved by the microbial feeding strategy during the bioprocess [8,9], by the generation of (nano)composites with diverse compatible materials [10,11], or by blending with suitable other polymers [11,12,13]. Importantly, the entire PHA production chain, encompassing the isolation of new robust productions strains, feedstock selection, fermentation technology, process engineering, bioreactor design, and, last but not least, downstream processing, needs to meet the criteria of sustainability [14]. The previously often-cited myth of biopolymers being per se more sustainable than established petrochemical plastics nowadays has finally been abandoned, as recently comprehensively reviewed; without conceiving the entire life cycle of biopolymers, it is impossible to conclude if they inherently outperform their petrochemical counterparts in terms environmental benefit [15].

Such economic, sustainability, and quality aspects are addressed in the 14 contributions to this special issue, “Advances in Polyhydroxyalkanoate (PHA) Production”. This issue evolved during a period of almost one year, and it was an outstanding pleasure for me to see so differently focused research groups participating in this mutual publication project. Authorships from 15 different countries from four continents were selected, having synergistic competences in diverse areas related to PHA research, some of them well-known in this scientific field already for years or even decades, and some of them currently attracting increasing attention in the scientific community for their recent research and development (R&D) achievements.

In principle, these contributions are dedicated to four major impact directions of PHA research: 

First, six articles deal with the assessment of inexpensive [16,17,18,19,20] or exotic [21] feedstocks to be used as carbon sources for PHA production. Importantly, these feedstocks constitute carbonaceous (agro)industrial waste streams, such as waste glycerol from biofuel production [16,17], lignocellulose waste from the food industry [18,20] and forestry [19], and even petrochemical plastic waste [21]. These works aim to find alternatives to commonly used feedstocks of value for human nutrition, in order to avoid the current “plate vs. plastic” dispute. Further, the articles show how such alternative feedstocks have to be pre-treated in order to minimize potential inhibitory effects on production strains.

Second, new insights into metabolic processes during intracellular PHA mobilization [22], metabolic flux analysis of PHA production by mixed microbial cultures (MMCs) [23], and novel molecular diagnostic techniques to trace new PHA production strains from diverse environments in a convenient manner [24] are presented.

Third, PHA production is introduced as an integral part of future (bio)refinery systems, as shown in the case of autotrophic PHA production by the effluent gases of a power plant [25], and the coupling of PHA biosynthesis with a wastewater treatment plant (WWTP) [26].

The fourth group of contributions addresses the fine-tuning of PHA composition on the monomeric level to facilitate its processing [27], and describes the processing of new PHA blends with other biocompatible materials to generate scaffolds for tissue engineering [28].

As the roof above all these articles, a comprehensive review makes us familiar with the current state of enhancing the sustainability, economics, and product quality of PHA [29]. The subsequent paragraphs intend to provide a short overview of the individual chapters of this special issue.

## 2. Individual Contributions

Kourmentza and colleagues provide a comprehensive review on current challenges and opportunities in PHA production. This article covers all hot spots during the multi-facetted PHA production lines. From the microbiological point of view, the application of both pure (monoseptic) cultures and MMCs is addressed. In the case of MMCs, the coupling of PHA to WWTPs is strongly encouraged by the authors. Special emphasis is also dedicated to raw material selection, process design, and the downstream processing for PHA recovery from microbial biomass. Regarding raw materials, the authors suggest abundant lignocelluloses as the future materials of choice to run a sustainable PHA production facility, and discuss recent advances in using toxic substrates like aromatic compounds, which would provide for bioremediation coupled to PHA biosynthesis. Moreover, halophile microbes are presented as stable production strains; their application should contribute to running PHA production processes at reduced sterility requirements. Finally, the outlook of this review refers to synthetic biology as a tool to achieve competitive PHA production by facilitating downstream processing, and to boost PHA productivity [29].

Takahashi and colleagues screened marine bacteria in order to assess their potential to thrive and accumulate PHA on the inexpensive substrate combination crude glycerol from the biodiesel industry and seawater. Out of 150 isolates, the authors report the identification of two auspicious new marine strains with high potential of PHA production on this substrate combination, which, in the future, should be subjected to detailed investigation and optimization in order to assess their applicability for industrial-scale PHA production [16].

In addition, Bhattacharya and associates used crude glycerol, in this case stemming from *Jatropha curcas*-based biodiesel production, as a carbon substrate for PHA production. Here, a Gram-positive production strain of marine origin, *Bacillus licheniformis* PL26, was used [17]. Such Gram-positive strains display the advantage of generating endotoxin-free PHA especially suitable for in vivo application in the biomedical field [30]. In addition to the intracellular storage product PHA, the authors also investigated the excretion of the extracellular product poly(ε-lysine), a material of significance inter alia for food preservation, by this organism. Regarding PHA production, the authors revealed that this organism accumulates the copolyester poly(3-hydroxybutyrate-*co*-3-hydroxyvalerate) (PHBHV) from waste glycerol as the sole carbon source without the need to add 3-hydroxyvalerate (3HV)-related precursor substrates [17].

Salgaonkar and Bragança investigated *Halogeometricum borinquense*, a new haloarchael PHA producer, as a PHA production strain on hydrolyzed sugarcane bagasse. Using this abundant lignocellulose substrate, *Hgm. borinquense* exhibited a superior performance in terms of PHA productivity and intracellular PHA fraction when compared to other scientifically rather undescribed haloarchaea, which were studied in parallel to this work. Furthermore, this strain was shown to produce a PHBHV copolyester from hydrolyzed bagasse without the need for precursor compounds [18], as also detected in the previous contribution [17]. Generally, this work contributes to the current quest for extremophile PHA producers, which are frequently described as the “rising stars” in the consortium of industrially production strains [31].

A similar substrate was used by Kucera and colleagues, who cultivated two *Burkholderia* strains (*B. cepacia* and *B. sacchari*) on the hydrolysate of spruce sawdust, a lignocellulosic wood waste. Sawdust hydrolysis was carried out both by applying strong acids to hydrolyze the hemicellulose fraction, and enzymatically to hydrolyze the cellulose fraction. This approach generates considerable amounts of fermentable sugars, which are converted by the two applied organisms towards biomass and PHA. Because this hydrolysate also contains growth-limiting components like polyphenols, furfural, acetic acid, or levulinic acid, the authors present a new, convenient upstream processing strategy to remove growth-inhibiting compounds from the hydrolysate by using inexpensive lignite (brown coal) instead of overliming or the commonly used, more expensive charcoal. As a further benefit, the authors suggest the value-added use of lignite after detoxification as an energy carrier [19].

Hokamura et al. used soybean wastewater from a Japanese *miso* production process for the accumulation of an intracellular PHA blend by a recombinant *Pseudomonas* sp. 61-3 harboring two different PHA synthase enzymes. For substrate preparation, soybean wastewater was spray-dried and used as a feedstock with and without subsequent hydrolysis. The intracellular blend consisted of poly(3-hydroxybutyrate) (PHB) homopolyester, and a randomly distributed copolyester consisting of 3-hydroxybutyrate (3HB) and longer 3-hydroxyalkanoates with four to 12 carbon atoms. Using hydrolyzed spray-dried soybean wastewater as the sole carbon source, the highest achieved concentration of this PHA blend amounted to 1 g/L, which corresponds to a PHA fraction of 35% of the cell dry mass. In this case, the blend contained about 80% 3HB and 20% longer building blocks, and displayed a flexible material with considerable toughness comparable to the characteristics displayed by poly(ethylene) (PE) [20].

Johnston and colleagues directly connected the utilization of petrol-based plastic waste with the production of PHA biopolymers. These authors used non-oxidized PE wax as an unconventional, exotic substrate for PHA production by the well-known eubacterial production strain *Cupriavidus necator* H16. In order to make this hydrophobic substrate accessible for the bacteria, the authors presented a viable sonication technique to produce an emulsion, which could be used as cultivation medium to thrive the bacteria. Non-oxidized PE wax displays the advantage of being conveniently produced from waste PE by simple thermal cracking and subsequent purification of the resulting gaseous stream; moreover, it has no other industrial use. Most of all, the authors underlined the independence of this substrate from food and feed applications, making the process ethically clear. In addition, it turned out that a PHBHV copolyester with abbot 11% 3HV in PHA is produced by *C. necator* when supplied with this substrate [15], which is similar to the findings for other new production strains in this special issue [17,18,20].

In contrast to the application of pure, monoseptic microbial cultures described in the above contributions, Montana-Herrera and co-workers used MMCs to study the monomeric composition of MMC-PHA during microbial growth and concomitant PHA accumulation. Different substrate feeding strategies using volatile fatty acids (VFAs) were investigated, showing a dynamic trend in biomass formation and monomeric PHA composition dependent on the substrate feed. Metabolic flux analysis was used to gain deeper insights into the goings-on in the MMC during the cultivation; revealing the correlation of reducing equivalents’ generation to the intracellular carbon flux, thus to the PHA composition on the monomeric level, which can be considered as a significant outcome of this study [24].

Karmann and colleagues focused their contribution on the investigation of population dynamics during medium chain length (*mcl*)-PHA production by *Pseudomonas putida* KT2440 at the level of individual cells under different environmental conditions. This work provides a completely new understanding of the mobilization of PHA during cell separation [22]. In contrast to the previously assumed paradigm, which suggested a balanced distribution of PHA granules to new daughter cells generated by binary division, the presented work teaches us that the distribution of PHA granules, often referred to as “carbonosomes” [32], under carbon-limited conditions occurs in an asymmetric manner; the culture segregates into a PHA-rich and a PHA-poor subculture, thus displaying a “bistable behavior” [22].

Morgana de Silva Montenegro and colleagues studied the microbial diversity of PHA-producing species by new molecular diagnosis techniques. These authors applied PHA synthase (*phaC*) by using suitable primers based on multiple alignments of PHA synthases from a total of 218 species with deciphered genomes for detecting new potential PHA producers; PHA synthases of type I and IV were used as positive controls to trace new organisms with PHA accumulation capacities. The authors describe the successful application of this new diagnostic technique to identify nine new marine PHA producers out of 16 marine isolates; when screening 37 additional isolates from different environments, about 30% among them were identified as potential PHA producers [23].

Pittmann and Steinmetz studied the production potential for PHA as a by-product of municipal WWTPs. Here, differently composed WWTP sludge lots were investigated as substrates under different operational conditions regarding pH-value, retention time, and withdrawal/refilling for optimized VFA production; short retention time and low withdrawal/refilling rate turned out to be the most beneficial for high VFA formation. In a second stage, generated VFA were used for high and stable production of PHA of constant monomeric composition in a feast/famine regime under fluctuating conditions. The authors suggest that this process, when using the entire quantity of sludge for all municipal WWTP in the European Union, could theoretically provide the feedstock for the production of about 20% of the current global PHA production [26].

Another concept for a PHA-based biorefinery was presented by Troschl and colleagues, who studied the solar-driven autotrophic pilot-scale cultivation of cyanobacteria for PHA production over extended periods in a 200-L horizontal tubular photobioreactor. As carbon source, CO_2_ from the gaseous effluents of an Austrian coal power plant was used. The authors describe the challenges they were confronted with during process development, and highlighted several issues considered as especially crucial for developing a stable cyanobacterial PHA production process, namely strain selection, CO_2_-availability, and process design and automation. Regarding strain selection, the authors suggest the use of rather small, unicellular cyanobacterial species like *Synechocystis* sp., which should be more resistant against shear stress when compared to the well-known filamentous cyanobacterial PHA-producer *Arthrospira* sp. [25].

Coming to defined applications of PHA, the authorship of Puppi et al. presented novel strategies to design new tissue engineering scaffolds by blending the copolyester poly(3-hydroxybutyrate-*co*-3-hydroxyhexanoate) (PHBHHx) with poly(ε-caprolactone) (PCL) as another biocompatible polymer. These blends were processed by the new method of “computer-aided wet-spinning”, a novel hybrid-additive manufacturing technique suitable for processing PHA in organic solution. The processing of PHA in solution instead of the thermal treatment normally used for PHA processing avoids adverse effects on PHA molecular mass. This technique successfully provided customer-made scaffolds with pre-defined architecture regarding macro- and micro-porosity. The high biocompatibility of these scaffolds was demonstrated by showing the successful adhesion and proliferation of pre-osteoblast cells on them, underlining their suitability for biomedical applications [28].

Poly(3-hydroxybutyrate-*co*-4-hydroxybutyrate) copolyesters with tailored 4-hydroxybutyrate (4HB) fraction, suitable for convenient processing, were produced in our laboratories in Graz, Austria, by Miranda de Sousa Dias et al. This was achieved by co-feeding the direct sucrose converter *B. sacchari* with sucrose from the Brazilian sugarcane industry and fine-tuned amounts of the 4HB-precursor γ-butyrolactone. The copolyesters were generated in fed-batch bioreactor setups at high productivity, and were subjected to detailed material characterization to evaluate their physicochemical properties and molecular mass distribution. As major outcome, it was proposed that the strain could act as one of the major industrial-scale PHA copolyester producers based on sucrose. As the conditio sine qua non for economic feasibility, the integration of the PHA production facilities into existing sugar production lines is necessary [27].

## 3. Conclusions

Global research efforts are currently devoted to the individual aspects needed to be addressed in order to facilitate the quick success of PHA-based materials on the polymer market. I hope that reading the *Bioengineering* special issue at hand will motivate and inspire researchers all over the world (and undergraduates interested in getting their feet on the ground of biopolymers!) to dedicate intensified efforts to further improve PHA production in terms of economics, product quality, and sustainability.
